# Analysis of circulating ceramides and hexosylceramides in patients with coronary artery disease and type II diabetes mellitus

**DOI:** 10.1186/s12872-023-03454-x

**Published:** 2023-09-12

**Authors:** Philip Düsing, Nadine N. Heinrich, Baravan Al-Kassou, Katharina Gutbrod, Peter Dörmann, Georg Nickenig, Felix Jansen, Andreas Zietzer

**Affiliations:** 1 Heart Center, Department of Internal Medicine II, University Hospital Bonn, Bonn, Germany; 2 Institute of Molecular Physiology and Biotechnology of Plants, University of Bonn, Bonn, Germany

**Keywords:** Coronary artery disease, Ceramides, Diabetes mellitus

## Abstract

**Background:**

Cardiovascular disease (CVD) remains the leading cause of death worldwide. The main driving force behind this association is coronary artery disease (CAD), the manifestation of atherosclerosis in the coronary circulation. Cornerstones in the development of CAD are pathologies in lipid metabolism. In recent years, ongoing research has identified ceramides, a subclass of sphingolipids to be mediators of CVD. The aim of this study is to investigate the influence of type II diabetes mellitus (DM) on circulating ceramides and hexosylceramides (HexCers) in CAD patients.

**Methods:**

24 patients aged 40–90 years with CAD confirmed by angiography were included into a pilot study. Patients with DM were identified by analysis of discharge letters or other medical documents available at the study center. During coronary angiography, arterial blood samples were collected and quantification of sphingolipids in patient serum was performed by mass spectrometry.

**Results:**

Statistical analysis showed nine significantly different HexCers in CAD patients with DM compared to patients without DM. Among the nine significantly regulated HexCers, we identified seven d18:1 HexCers. This group contributes to the fourth most abundant subgroup of total ceramides and HexCers in this dataset. HexCer-d18:1–23:1(2-OH) showed the strongest downregulation in the patient group with DM.

**Conclusion:**

This study suggests that levels of circulating HexCers are downregulated in patients with CAD and concomitant DM compared to patients without DM. Further research is needed to investigate the underlying mechanisms and the suitability of HexCers as possible mediators and/or prognostic markers in CAD.

**Supplementary Information:**

The online version contains supplementary material available at 10.1186/s12872-023-03454-x.

## Background

Cardiovascular disease (CVD) remains the leading cause of death worldwide. The main driving force behind this association is coronary artery disease (CAD), the manifestation of atherosclerosis in the coronary circulation [1, 2]. CAD is a chronic condition which is characterized by inflammation, endothelial dysfunction and plaque formation within the coronary arteries [2, 3]. Diabetes mellitus (DM) is well known to be a highly aggressive cardiovascular risk factor that leads to a two-fold increased risk for development of CAD [2]. Among different types of DM, type II accounts for the majority of cases [4]. The prevalence of DM is expected to continue to grow in parallel with increasing rates of unhealthy lifestyle, obesity and overnutrition in western and developing countries [4]. Cornerstones in the development of CAD are pathologies in lipid metabolism which is an ongoing focus of clinical and experimental research. A recently published study investigating the genetic and molecular profile of the adiponectin metabolic pathway in diabetic dyslipidemia found a complex interplay of genetic and biochemical parameters in diabetic dyslipidemia, which is significant from the perspective of risk stratification and novel therapeutic strategy development [5]. In recent years, research has identified ceramides, a subclass of sphingolipids to be mediators of CVD [6]. Ceramides can be produced by all cell-types by de-novo synthesis, via the sphingomyelinase, or by the catabolic/salvage pathway [7]. Ceramides play a role in several physiological processes such as regulating vascular tone [8]. Observational studies have identified certain circulating ceramide subspecies containing C16.0 and C18.0 acyl groups to be associated with increased cardiovascular risk [9, 10]. Based on these studies, ceramide risk scores have been developed for clinical assessment of cardiovascular risk which showed promising results in a large community based cohort [11]. On a cellular level, ceramides have been linked to pathophysiological hallmarks of atherosclerosis such as endothelial dysfunction. Interestingly, ceramides were shown to promote endothelial apoptosis under hyperglycemic conditions [12, 13]. Ceramides have been shown to be involved in development of insulin resistance, a crucial pathophysiological feature of type II DM [14]. Furthermore, pharmacological inhibition of serine palmitoyl transferase which is crucial for sphingolipid de novo synthesis is able to prevent insulin resistance in mice [15]. These findings suggest that ceramide metabolism is involved in the pathophysiology of DM and may also promote CVD.

Thus, ceramides are suitable as circulating diagnostic markers and may represent targets for pharmacological therapies. However, information on the regulation of ceramide levels in patients with DM and established CAD are lacking. The ceramide metabolism is central for sphingolipid synthesis and degradation and it is involved a complex network of enzymes, in which ceramide molecules can be modified on a molecular level [7]. This results in different ceramide-derived sphingolipids with distinct biological features. One example is the enzymatic addition of a hexose molecule (glucose or galactose) resulting in complex sphingolipids named hexosylceramides (HexCers) [16]. While the biological function of highly abundant, unmodified ceramides has already been described, the role of less abundant, HexCers has been insufficiently investigated with respect to cardiovascular disease [16]. The aim of the present study is to investigate the influence of type II DM on plasma-levels of high and low-abundant ceramides and HexCers in patients with CAD.

## Methods

### Study population

**Fig. 1:**
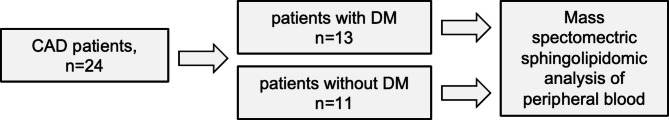
Flowchart of the Study protocol

Patients that underwent routine coronary angiography between January and May 2019 at the Heart Center Bonn provided written informed consent to be included into a pilot study. A retrospective analysis was performed to identify eligible patients. Inclusion criteria were CAD which was confirmed during angiography and an age between 40 and 90 years. Patients with type II DM were identified by analysis of discharge letters or medical charts available at the study center. Prevalence of cardiovascular risk factors as well as information about pharmacological therapies were analyzed from discharge letters. Patients with CAD and without type II DM represent the control group (Fig. 1). Exclusion criteria were severe liver disease, inflammatory or malignant disease, potential pregnancy, leukopenia, thrombocytopenia, and psychotic disease.

### Blood sample collection and preparation

During coronary angiography, arterial blood samples were collected under sterile conditions via the femoral or radial sheath. For sphingolipid analyses, a total of 1.5 ml was transferred in an EDTA tube. Blood samples were centrifuged in three cycles at 4 °C for 15 min with 3000 g to collect plasma for further analyses.

### Mass spectrometric quantification of sphingolipids

Quantification of sphingolipids in patient serum was performed by using a QTRAP 6500 + LC–MS/MS system (Sciex, Darmstadt), as described previously [17].

### Statistics

Statistical analyses were performed with the software Prism9. Statistical details are displayed in the figure legends. For multiple testing, mass spectrometric analysis of patients were analyzed by Mann-Whitney U test to compare differences between patients with DM and without DM. As post hoc analysis, a false discovery rate (FDR) approach was performed by using two-stage step-up method of Benjamini, Krieger and Yekutieli with a desired q-value of 5%.

## Results

### Patient characteristics

Overall, a total of 24 patients were enrolled in the study. 13 patients had established type II DM and 11 patients without DM served as a control group. Patient characteristics are displayed in Table 1. Mean age in the DM cohort was 69.3 years vs. 72.2 years in the control group. According to the study design, all patients were diagnosed with CAD during coronary angiography. In the DM group, more patients were treated with percutaneous coronary intervention during coronary angiography (100.0% vs. 72.7%). Furthermore, patients with DM showed a higher prevalence of cardiovascular risk factors such as hypertension (84.6% vs. 63.6%) or chronic kidney disease (69.2% vs. 36.4%), whereas patients without DM showed a higher prevalence of dyslipidemia (84.6% vs. 90.9%).

**Table 1:** Patient characteristics. CAD; coronary artery disease, DM; diabetes mellitus

Patient characteristics	Type II DM (n = 13)	No DM (n = 11)	Total (n = 24)
Age – years (mean ± SD)	69.3 ± 13.1	72.2 ± 10.3	70.6 ± 1.7
Male sex – no. (%)	9 (69.2)	8 (61.5)	17 (70.8)
Coronary artery disease – no. (%)	13 (100.0)	11 (100.0)	24 (100.0)
1 vessel CAD – no. (%)	1 (7.7)	2 (18.2)	3 (12.5)
2 vessel CAD – no. (%)	5 (38.5)	3 (27.3)	8 (33.3)
3 Vessel CAD – no. (%)	7 (53.9)	6 (54.6)	13 (54.2)
Percutaneous coronary intervention during coronary angiography – no. (%)	13 (100.0)	8 (72.7)	21 (87.5)
Left ventricular ejection fraction in ventriculography			
> 50%	10 (76.9)	7 (63.6)	17 (70.8)
40–49%	2 (15.4)	1 (9.1)	3 (12.5)
< 40%	1 (7.7)	3 (27.3)	4 (18.2)
Cardiovascular risk factors			
Type II DM – no. (%)	13 (100.0)	0 (0)	13 (54.2)
Arterial hypertension – no. (%)	11 (84.6)	7 (63.6)	18 (75.0)
Dyslipidemia – no. (%)	11 (84.6)	10 (90.9)	21 (87.5)
Smoking status			
Current smoker – no. (%)	2 (15.4)	2 (18.2)	4 (18.2)
Quitter – no. (%)	5 (38.4)	3 (27.3)	8 (33.3)
No smoker – no. (%)	6 (46.2)	6 (54.6)	12 (50.0)
Chronic kidney disease – no. (%)	9 (69.2)	4 (36.4)	
Antidiabetic medication at discharge			n = 23
None	2 (15.4)	11 (100.0)	13 (56.5)
Insulin	2 (15.4)	0 (0)	2 (8.7)
Metformin	8 (61.5)	0 (0)	8 (34.8)
Gliptine	5 (38.4)	0 (0)	5 (21.7)
Sulfonylurea	1 (7.7)	0 (0)	1 (4.4)
SGLT2-Inhibitor	1 (7.7)	0 (0)	1 (4.4)
Lipid lowering therapies at discharge			
Statin	11 (92.0)	11 (100.0)	22 (95.7)
Ezetemib	1 (8.3)	1 (9.1)	2 (8.7)
PCSK9-Inhibitors	1 (8.3)	0 (0)	1 (4.4)

### Sphingolipid analysis in patient serum

**Fig. 2:**
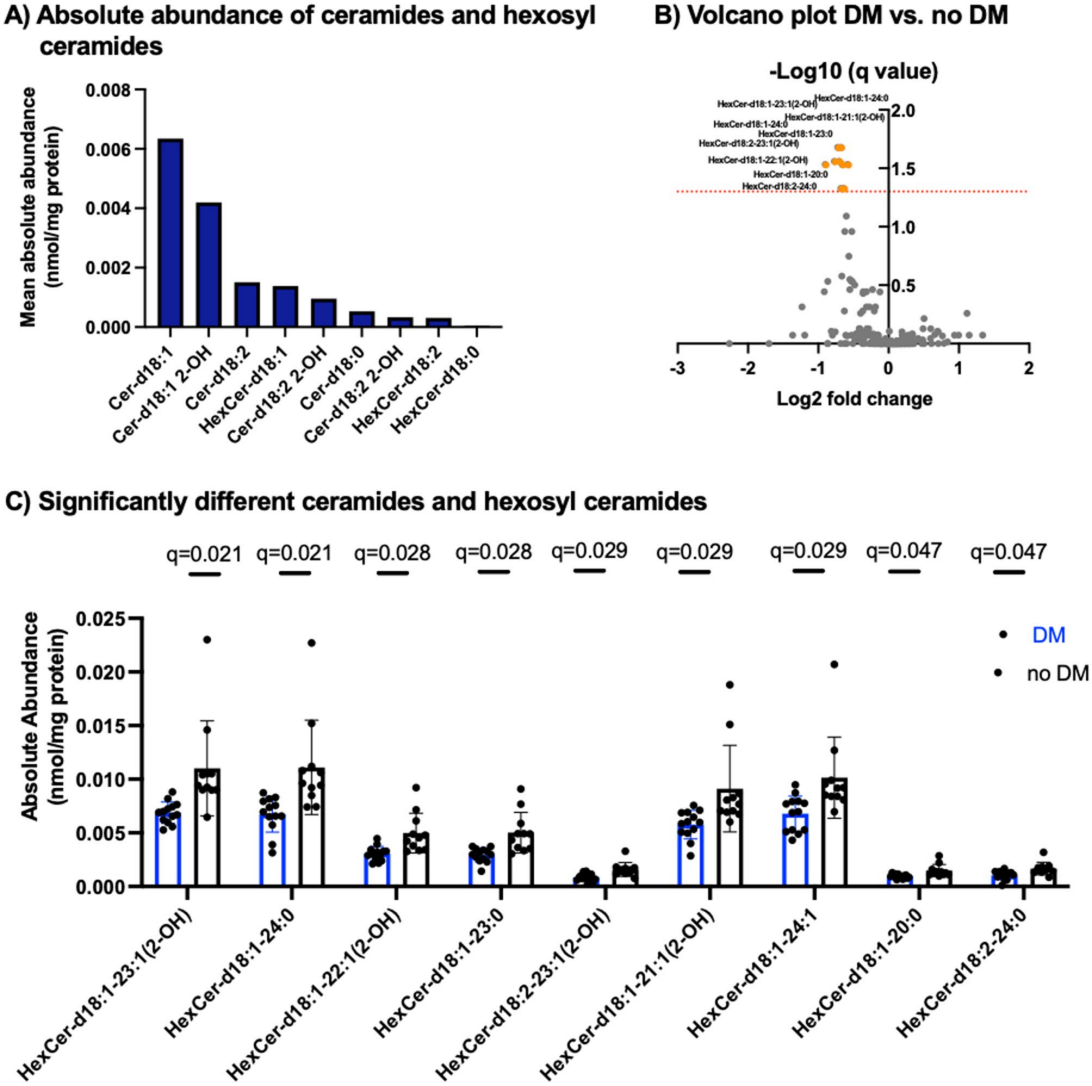
**(A)** Mean absolute abundance of different molecular species of ceramides and HexCers in CAD patients. **(B)** Volcano plot of sphingolipid analysis of patients with DM vs. no DM. **(C)** Absolute abundance of significantly regulated sphingolipids between patients with DM and without DM. Data are displayed as mean ± SD. CAD, coronary artery disease; DM, diabetes mellitus; FDR, false discovery rate; SD, standard deviation

We first evaluated our dataset regarding the absolute abundance of single molecular species of ceramides, as a high abundance may be an indicator for biological relevance of these molecules. Quantitative analysis revealed that d18:1 ceramide are the most abundant molecular species of ceramides in our dataset (Fig. 2A). Statistical analysis showed that a total of 9 molecular species of HexCers (Fig. 2C) were found in significantly lower plasma concentrations in patients with DM compared to patients without DM (Fig. 2B). These analyses demonstrate a significant downregulation of HexCers in CAD patients with DM compared to patients without DM. Among the 9 significantly regulated HexCers, we identified 7 d18:1 HexCers that contribute to the fourth most abundant subgroup of ceramides and HexCers in our dataset. HexCer-d18:1–23:1(2-OH) showed the strongest downregulation under the influence of DM.

## Discussion

In this study, we investigated the influence of DM on circulating ceramides and HexCers in CAD patients with a high resolution lipidomic approach. Our findings demonstrate that DM is associated with a lower levels of circulating HexCers in the peripheral blood of CAD patients.

HexCers are a class of glycosphingolipids. Their structure is characterized through a hydrophilic hexose (glucose or galactose) as well as a hydrophobic ceramide [16]. Glycosylation, i.e. the transfer of a glucose or galactose moiety, is catalyzed by the enzymes UDP-glucose:ceramide glucosyltransferase (GlcCer-synthase) or UDP-galactose:ceramide galactosyltransferase (GalCer-synthase), respectively [16]. Both enzymes are ubiquitously expressed in mammalian tissue [16, 18]. Glycosphingolipids are involved in numerous physiological processes such as cell proliferation and inflammation [16]. Pathologic alteration of these processes are cornerstones in the pathophysiology of atherosclerosis and thereby might be potential molecular mechanisms connecting CAD with altered HexCers metabolism [3, 19]. The important physiological role of HexCers is highlighted by the fact that genetic knockdown of GlcCer-synthase in a murine model is lethal at an embryonic stage [16]. This may be due to the fact, that GlcCers represent the basis for the synthesis of a large number of glycosphingolipids, which, on a cellular level, are involved in the regulation of differentiation, proliferation and cell growth [20]. Moreover, HexCers are biologically active molecules that play a crucial role in a number of physiological processes. Experimental data suggest, that pharmacologic inhibition of GlcCer-synthase leads to a reduction of proliferation capability of renal epithelial cells [21]. Furthermore, RNA-interference of GlcCer-synthase induces apoptosis in embryonal cells of *Drosophila melanogaster* [22]. Potential mechanisms for these pro-proliferative and anti-apoptotic effects of HexCers might include an alteration of the intracellular “ceramide-pool”. A reduced synthesis of GlcCers was shown to lead to an increased synthesis of other ceramide-derived lipids, which might promote pathophysiological processes [16, 22, 23]. To date, only few studies have investigated the influence of pathological glucose metabolism on circulating HexCers. Thus, in a murine model of DM, hyperglycaemic conditions induced a higher concentration of HexCers in explanted tissue of the adrenal cortex [24]. In another study using ex vivo human tissue, no difference could be observed in HexCers concentration observed in ocula vitreous bodies between patients with and without DM [25].

Limitations of the present study are its small sample size and the retrospective, observational design. Further, we cannot conclude any mechanistic effect of DM on circulating HexCers levels in the peripheral blood. Potential confounders of the observed effects such as potential differences in the characteristics of the two patient groups or selection bias also remain. Further, the study was only conducted in CAD patients and does not allow conclusions for patients without CAD.

## Conclusion

Despite these limitations, our study suggests that levels of circulating HexCers may be downregulated in patients with CAD and concomitant DM compared to patients without DM. Therefore this study may represent the foundation for future investigations of mechanisms of circulating HexCers in diabetic patients with CAD. These studies may lead to more insights to use circulating HexCers as diagnostic markers to identify high risk individuals among CAD patients. Future studies should cover both mechanistic investigations in cell culture and animal models and investigating clinical outcomes in a larger cohort of patients with altered levels of HexCers. Furthermore, advanced mechanistic insights in HexCers metabolism under pathologic conditions might identify potential therapeutic targets and novel pharmacological approaches.

### Electronic supplementary material

Below is the link to the electronic supplementary material.

Additional File 1: Masspectrometric Analysis of Ceramides and HexCeramides in Patient with and without DM

## Data Availability

The datasets used and/or analysed during the current study available from the corresponding author on reasonable request.

## References

[1] World Health Organization. The top 10 causes of death. 2020. https://www.who.int/news-room/fact-sheets/detail/the-top-10-causes-of-death. Accessed on 2022-02-09.

[2] Knuuti J, Wijns W, Saraste A, Capodanno D, Barbato E, Funck-Brentano C (2020). 2019 ESC Guidelines for the diagnosis and management of chronic coronary syndromes. Eur Heart J.

[3] Ross R. Atherosclerosis — An Inflammatory Disease. Epstein FH, editor. N Engl J Med. 1999;340(2):115–26. 10.1056/NEJM199901143400207 9887164

[4] Dal Canto E, Ceriello A, Rydén L, Ferrini M, Hansen TB, Schnell O (2019). Diabetes as a cardiovascular risk factor: an overview of global trends of macro and micro vascular complications. Eur J Prev Cardiol.

[5] Ghoshal K, Chatterjee T, Chowdhury S, Sengupta S, Bhattacharyya M (2021). Adiponectin genetic variant and expression coupled with lipid peroxidation reveal New Signatures in Diabetic Dyslipidemia. Biochem Genet.

[6] Choi RH, Tatum SM, Symons JD, Summers SA, Holland WL (2021). Ceramides and other sphingolipids as drivers of cardiovascular disease. Nat Rev Cardiol.

[7] Zietzer A, Düsing P, Reese L, Nickenig G, Jansen F (2022). Ceramide Metabolism in Cardiovascular Disease: A Network with High Therapeutic potential. Arterioscler Thromb Vasc Biol.

[8] Cogolludo A, Villamor E, Perez-Vizcaino F, Moreno L (2019). Ceramide and Regulation of Vascular Tone. Int J Mol Sci.

[9] Gencer B, Morrow DA, Braunwald E, Goodrich EL, Hilvo M, Kauhanen D (2022). Plasma ceramide and phospholipid-based risk score and the risk of cardiovascular death in patients after acute coronary syndrome. Eur J Prev Cardiol.

[10] Hilvo M, Meikle PJ, Pedersen ER, Tell GS, Dhar I, Brenner H et al. Development and validation of a ceramide- and phospholipid-based cardiovascular risk estimation score for coronary artery disease patients. Eur Heart J. 2019;ehz387. 10.1093/eurheartj/ehz387 31209498

[11] Vasile VC, Meeusen JW, Medina Inojosa JR, Donato LJ, Scott CG, Hyun MS (2021). Ceramide Scores Predict Cardiovascular Risk in the community. Arterioscler Thromb Vasc Biol.

[12] Zietzer A, Jahnel AL, Bulic M, Gutbrod K, Düsing P, Hosen MR (2022). Activation of neutral sphingomyelinase 2 through hyperglycemia contributes to endothelial apoptosis via vesicle-bound intercellular transfer of ceramides. Cell Mol Life Sci.

[13] Luo Y, Lei M (2017). α-Mangostin protects against high-glucose induced apoptosis of human umbilical vein endothelial cells. Biosci Rep.

[14] Chaurasia B, Tippetts TS, Mayoral Monibas R, Liu J, Li Y, Wang L (2019). Targeting a ceramide double bond improves insulin resistance and hepatic steatosis. Science.

[15] Holland WL, Brozinick JT, Wang LP, Hawkins ED, Sargent KM, Liu Y (2007). Inhibition of Ceramide Synthesis ameliorates Glucocorticoid-, Saturated-Fat-, and Obesity-Induced insulin resistance. Cell Metab.

[16] Ishibashi Y, Kohyama-Koganeya A, Hirabayashi Y (2013). New insights on glucosylated lipids: metabolism and functions. Biochim Biophys Acta BBA - Mol Cell Biol Lipids.

[17] Woeste MA, Stern S, Raju DN, Grahn E, Dittmann D, Gutbrod K (2019). Species-specific differences in nonlysosomal glucosylceramidase GBA2 function underlie locomotor dysfunction arising from loss-of-function mutations. J Biol Chem.

[18] Gault CR, Obeid LM, Hannun YA. An Overview of Sphingolipid Metabolism: From Synthesis to Breakdown. In: Chalfant C, Poeta MD,Sphingolipids as Signaling and Regulatory Molecules [Internet]. New, York. NY: Springer New York; 2010 [cited 2022 Jun 20]. p. 1–23. (Back N, Cohen IR, Lajtha A, Lambris JD, Paoletti R, editors. Advances in Experimental Medicine and Biology; vol. 688). Available from: http://link.springer.com/10.1007/978-1-4419-6741-1_1. 10.1007/978-1-4419-6741-1_1 PMC3069696 20919643

[19] Falk E (2006). Pathogenesis of atherosclerosis. J Am Coll Cardiol.

[20] Hannun YA, Obeid LM (2018). Sphingolipids and their metabolism in physiology and disease. Nat Rev Mol Cell Biol.

[21] Shayman JA, Deshmukh GD, Mahdiyoun S, Thomas TP, Wu D, Barcelon FS (1991). Modulation of renal epithelial cell growth by glucosylceramide. Association with protein kinase C, sphingosine, and diacylglycerol. J Biol Chem.

[22] Kohyama-Koganeya A, Sasamura T, Oshima E, Suzuki E, Nishihara S, Ueda R (2004). Drosophila Glucosylceramide Synthase J Biol Chem.

[23] Reza S, Ugorski M, Suchański J (2021). Glucosylceramide and galactosylceramide, small glycosphingolipids with significant impact on health and disease. Glycobiology.

[24] Subathra M, Korrapati M, Howell LA, Arthur JM, Shayman JA, Schnellmann RG (2015). Kidney glycosphingolipids are elevated early in diabetic nephropathy and mediate hypertrophy of mesangial cells. Am J Physiol-Ren Physiol.

[25] Wilmott LA, Grambergs RC, Allegood JC, Lyons TJ, Mandal N (2019). Analysis of sphingolipid composition in human vitreous from control and diabetic individuals. J Diabetes Complications.

